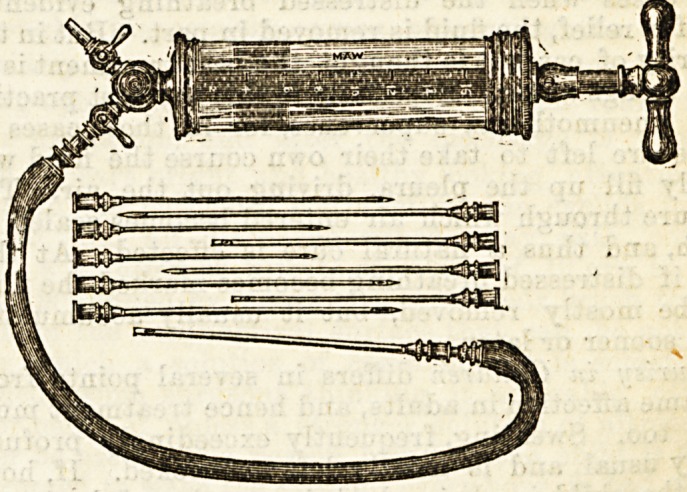# Treatment of Pleurisy

**Published:** 1893-03-25

**Authors:** 


### The Hospital Clinic.

[*The Editor will be glad to receive offers of co-operation and contributions from members of the profession All letters should be addressed to* The Editor, The Lodge, Porchester Square, London, W.]

### ROYAL HOSPITAL FOR DISEASES OF THE CHEST, CITY ROAD, E.C.

### Treatment of Pleurisy.

The history of the treatment of pleurisy is in itself a brief history of medicine, showing the oscillations of opinion between interference and non-interference, and it is really only within the last twenty years that the question has been settled, as to whether paracentesis should be performed, and if so, at what period of the disease. In Trousseau's time, thirty years ago, the debate raged fiercely, and he himself was bitterly attacked by those who, on very feeble evidence, had adopted the view that pleurisy was never fatal, and that if the effusion was let alone recovery was certain. But Trousseau's sound views, based on extensive clinical experience and accurate observation, gained the day, and it is to him, more than any other man, that we owe our present practice.

In considering the subject of the treatment of the disease at the City Road Chest Hospital, it will be well for the sake of clearness to classify the different clinical forms that it may assume. In the first place a broad distinction may be drawn between pleurisy as found in adults, and as found in children. Taking adults first, the cases fall under the following heads: 1. Dry Pleurisy. 2. Pleurisy with serous effusion. 3. Pleurisy with purulent effusion. 4. Pleurisy in Phthisis.

1. *Dry Pleurisy* apart from any other disease is not a common form; that is to say, it is not usual for the pleurisy to remain dry. If, however, the disease is caught in an early stage, it is possible sometimes to abort it, so to speak, and, by careful treatment, to prevent effusion. It is at first astonishing to observe how quickly the pain, even if very severe, described as like a dagger being plunged into the side, is relieved by carefully strapping the affected side. Strips of Leslie's or similar strapping are applied, each strip being about one and a half or two inches in width, and they should reach from the opposite border of the sternum to about two inches across the spines of the vertebræ on to the sound side. When five or six strips have been applied, each overlapping the one below, two vertical pieces are put to secure the ends. Instead of strapping, blisters are sometimes preferred; if, for example, breathing is at all weak, or if there be any heart trouble. Either blistering fluid or a useful American blistering plaster, "Canthos," are the favourite means employed, and with these a number of blisters can be produced, one after the other, waiting till one has well risen before the next is put on at some contiguous spot. Unless the breathing is clearly distressed, and the temperature much raised (101° or more) the patient is not kept in bed, and in fact many cases occur amongst the out-patients and never come into the wards at all. Of medicines the Hospital Pharmacopœial mist. amm. acetat. co. (liq. amm. acet. ʒss., sp. æth. nitr. ♏xxx. syr. limon. ʒi., aq. camph. ad. ℥i.) is very useful, as also is mist. ammon. et chlorof. (ammon. carb. gr. iv.) and mist. quin. et pot. iod. (quin. sulph. gr. i., pot. iod. gr. v.). Cough, if troublesome, can be met by the first or second of these mixtures, or by linctus scillæ c. opio. (oxymel. scill. ♏xx., tr. camph. co. ♏x., vin. ipecac. ♏v., mucilag. acac. ad. ʒi.), or troch. glycyrrh (containing extr. glycyrrh and ol. anisi.).

2. *Pleurisy with Serous Effusion* is by far the commonest form of the affection which has to be treated in the hospital, but here again it is not rare for out-patients suffering in this way to do quite well without requiring to be taken in. Of course the aim of our treatment is to promote the speedy absorption of the fluid, and for this purpose the local application of a mixture of equal parts of the liniment and tincture of iodine is the usual practice. This has the further advantage of relieving pain if that be present. Of internal remedies mist.quin.c.pot.iod.(vid.supr.) is commonly used, or mist. pot. acet. co. (pot. acet. gr. xx., pot. nitr. gr. v., sp. aeth. nitr. ♏xxx., acet. scill. ♏x., liq. ammon. acet. ʒij., aq. camph. ad. ℥i). With the latter of these it is not uncommon to combine tr. digitalis ♏. v.—x. If pain be persistent, which is not usual, the side is strapped or blistered, and sleep is procured by small doses of morphia, hypodermically or by the mouth.

In many cases these measures suffice, and the effusion gradually clears up, but in a certain proportion the contrary takes place, and the physical signs indicate that day by day there is an increasing quantity of fluid in the pleura. On an average the accumulation does not become sufficient to cause much dyspnœa till three weeks or so have elapsed, and it is the general practice to allow that time to pass before paracentesis is resorted to. But should symptoms become severe before the end of three weeks the fluid is removed at once. The exploring syringe used at the City Road Hospital is of the shape shown in the figure, which enables the fluid to be withdrawn as soon as found without the necessity for taking the needle out and substituting an aspirating needle. The indiarubber tube attached to the syringe is connected to a longer indiarubber tube previously filled with water containing carbolic acid (1 in 40), and the fluid is kept running by the syphon action of this longer tube. When it is judged that about two-thirds of the effusion has been drawn off the stop-cocks are closed and the needle withdrawn. It is generally found that absorption of the remaining fluid goes on rapidly after this operation. The spot chosen for puncturing the chest depends to some extent on the amount of fluid present, but commonly it is in the mid axillary line in the sixth interspace, or else at a spot midway between the positions occupied by the point of the inferior angle of the scapula when the arm is at the side, and when it is raised up to the side of the head. The needle being withdrawn, the puncture is covered by a scrap of absorbent wool and some collodion, over which a small piece of lint is placed and fixed by strapping, cut gridiron fashion.

The patient is kept in bed on a light diet, with a limited quantity of fluid, about one pint of milk a day with a small amount of soda water if desired. Convalescence is usually rapid, and in from two to three weeks the patient is able to leave the ward.

It may be worth noting that the exploring syringe is not generally used for purposes of diagnosis, unless there is some doubt as to whether there really is any effusion, or as to whether it is serum or pus. Thus we are spared the annoyance, not infrequent in some cases, of seeing a serous effusion converted into a purulent one.

(3) *Pleurisy with Purulent Effusion* will not be dealt with in this paper, as it would lead us too far.

(4) *Pleurisy in Phthisis* may be either dry or with effusion. The former is probably the more common, judging from what one sees on the post-mortem table. The chief symptom, of course, is pain, and this, if slight, is often relieved by the application of two or three hot fomentations. If of severer character, strapping is usually sufficient, but it must be put on tightly and kept on for a week or ten days. This, of course, cannot be well managed at the apices of the lungs, and pain in these spots is best treated by blisters or by iodine, used as before described, and painted over patches of about two inches square. Thus, in the course of a few days, by changing the place of application the whole of the apex back and front may be treated, and not only is pain relieved, but the application is of great use as a counter irritant in stopping the tubercular growth in the lungs. Crepitations disappear, and the mischief is evidently held in check. This, of course, applies only to early stages or to chronic cases.

With effusion, the usual treatment is—do nothing. The lung is kept at rest, is more or less collapsed; hence has reduced blood supply, and the tubercular processes seem to be arrested. There are, of course, exceptions to this, as when the sounder lung of the two in double phthisis becomes affected by pleurisy, and in such cases when the distressed breathing evidently calls for relief, the fluid is removed in part. But in the majority of cases it is thought the best treatment is to leave things alone. Especially is this the best practice when pneumothorax supervenes, for in these cases if events are left to take their own course the fluid will usually fill up the pleura, driving out the air. The aperture through which air entered becomes sealed by lymph, and thus a natural cure is effected. At this stage if distressed breathing becomes marked the fluid may be mostly removed, but it usually accumulates again sooner or later.

*Pleurisy in Children* differs in several points from the same affection in adults, and hence treatment must differ too. Sweating, frequently exceedingly profuse, is very usual, and is mostly left unchecked. If, however, the child is obviously being weakened by it, the pill of zinc and belladonna (zinc. oxidi, gr. iij., ext. bellad., gr. ¼, ext. hyoscy., gr. j.) is given every night, or a pill containing atropine gr. 1-200. Pain is often very severe, and hot fomentations are of great service in relieving this symptom; if these are insufficient, dry cupping is employed. The diuretic and sudorific mixtures mentioned previously are given in smaller doses, and together with the application of iodine, usually effect a cure. Children as a rule show signs of dyspnœa sooner than adults, and hence puncture is generally employed earlier. The cardinal symptoms showing the necessity for this are displacement of the mediastinum, and orthopnœa.,

The greater frequency of empyema in children must be borne in mind, even without much rise in temperature, but the treatment of this form of pleurisy does not come within the scope of this account.

## Figures and Tables

**Figure f1:**